# Characterizing mammographic images by using generic texture features

**DOI:** 10.1186/bcr3163

**Published:** 2012-04-10

**Authors:** Lothar Häberle, Florian Wagner, Peter A Fasching, Sebastian M Jud, Katharina Heusinger, Christian R Loehberg, Alexander Hein, Christian M Bayer, Carolin C Hack, Michael P Lux, Katja Binder, Matthias Elter, Christian Münzenmayer, Rüdiger Schulz-Wendtland, Martina Meier-Meitinger, Boris R Adamietz, Michael Uder, Matthias W Beckmann, Thomas Wittenberg

**Affiliations:** 1 University Breast Center for Franconia, Erlangen-Nuremberg Comprehensive Cancer Center, Erlangen University Hospital, Department of Gynecology and Obstetrics, Universitaetsstrasse 21-23, 91054 Erlangen, Germany; 2 Fraunhofer Institute for Integrated Circuits IIS, Am Wolfsmantel 33, 91058 Erlangen, Germany; 3 International Max Planck Research School (IMPRS) for Optics and Imaging, Erlangen, Germany; 4 Department of Medicine, Division of Hematology and Oncology, David Geffen School of Medicine, University of California at Los Angeles, USA; 5 Institute of Diagnostic Radiology, Erlangen University Hospital, Erlangen-Nuremberg Comprehensive Cancer Center, Universitaetsstrasse 21-23, 91054 Erlangen, Germany

## Abstract

**Introduction:**

Although mammographic density is an established risk factor for breast cancer, its use is limited in clinical practice because of a lack of automated and standardized measurement methods. The aims of this study were to evaluate a variety of automated texture features in mammograms as risk factors for breast cancer and to compare them with the percentage mammographic density (PMD) by using a case-control study design.

**Methods:**

A case-control study including 864 cases and 418 controls was analyzed automatically. Four hundred seventy features were explored as possible risk factors for breast cancer. These included statistical features, moment-based features, spectral-energy features, and form-based features. An elaborate variable selection process using logistic regression analyses was performed to identify those features that were associated with case-control status. In addition, PMD was assessed and included in the regression model.

**Results:**

Of the 470 image-analysis features explored, 46 remained in the final logistic regression model. An area under the curve of 0.79, with an odds ratio per standard deviation change of 2.88 (95% CI, 2.28 to 3.65), was obtained with validation data. Adding the PMD did not improve the final model.

**Conclusions:**

Using texture features to predict the risk of breast cancer appears feasible. PMD did not show any additional value in this study. With regard to the features assessed, most of the analysis tools appeared to reflect mammographic density, although some features did not correlate with PMD. It remains to be investigated in larger case-control studies whether these features can contribute to increased prediction accuracy.

## Introduction

Mammographic density (MD) is an important risk factor for breast cancer. Consistent evidence has emerged during the last 10 years that women with a high MD have a twofold to fivefold increase in risk in comparison with women with a low MD [1-3].

Several methods of measuring MD have been described. Subjective methods include Wolfe patterns, with four categories [4,5]; Boyd classification, with six categories [6]; and subjective assessment of the percentage density by a reader, with values between 0 and 100% [7]. In addition to these completely subjective methods, several computer-assisted methods have been developed, such as *Madena *and *Cumulus *[8-10]. Specifically, these computer programs assess MD as the proportion of the area with dense breast tissue in relation to the whole breast area on a mammogram. These methods have served to date as the gold standard for assessing the percentage mammographic density (PMD).

Despite these technologic advances, however, interobserver and intraobserver variability continue to be important and as yet unresolved issues. Automated computer measurement of MD and standardization of digital mammograms for automated analysis have been investigated in some studies [11,12]. These methods mimic the subjective assessment of MD. A method using fully automated analysis of texture patterns in the mammogram might be able to assess and characterize digital or digitized mammograms and reveal additional textural features. These might help differentiate between breast cancer patients and healthy controls.

Several hundred textural features and variants have been developed and proposed during the last few decades for various applications in the field of biomedical image processing, including the characterization of mammographic lesions for diagnostic purposes [13-18]. Textural features have also been investigated in relation to distinguishing between mammograms of breast cancer patients and controls [19]. These features can be broadly grouped into statistical, moment-based, form-based, structural, and spectral features. A detailed description of each feature group is given in the Methods section.

The aim of this study was to evaluate a variety of automated texture features as risk factors for breast cancer, by using a case-control study design. In addition, the textural-feature analysis was to be compared with semiautomatically assessed PMD.

## Materials and methods

### Study population and assessment of percentage mammographic density

The basis for this analysis was provided by a case-control study (the Bavarian Breast Cancer Cases and Controls), which was designed to investigate genetic risk factors and prognostic factors for breast cancer [20,21], and which is part of the Breast Cancer Association Consortium [22-24]. Mammographic density also was assessed in the cases and controls, as reported elsewhere [25]. In brief, the cases included in the study were hospital based and age matched with population-based controls from 2004 and 2005. The cases were incident cases and were referred to the breast center either by physicians after an early-detection examination or by themselves. No population-based screening program existed in this area at that time. The participants completed a questionnaire providing epidemiologic data during an interview to obtain information about common epidemiologic risk factors, such as hormone replacement therapy, body mass index, and family medical history.

All of the women included provided written informed consent for participation in the study, and the ethics committee of the University of Erlangen-Nuremberg, Germany, approved the research project.

Analogue and film printouts of digital mammograms were scanned and digitized by using the CAD PRO Advantage film digitizer (VIDAR Systems Corporation, Herndon, VA, USA), and the percentage mammographic density was assessed by using the Madena software program, version X (Eye Physics, LLC, Los Alamitos, CA, USA) [8].

For the present investigation, the digitized mammograms from the study were analyzed by using automated image texture analysis. More precisely, the image texture analysis was performed on the delineated breast area, which is termed region of interest (ROI). Only craniocaudal and contralateral images for the cases and mammograms without lesions for the controls were used for the analysis, and scans of film printouts from the digital mammograms were treated in the same way as analogue ones. Characteristic image texture measures were computed and analyzed for a total of 864 cases and 418 controls. In all, 636 of the cases (74%) and 213 of the controls (51%) had analogue mammograms; all of the others were digital.

### Semiautomated delineation of the breast area

A four-step algorithm for delineating the breast was developed to automate the process of image analysis of the breast tissue on the digital and digitized mammograms. In the first step, white stripes close to the image border are eliminated. After that, an adapted version of the Otsu thresholding algorithm [26] is used to separate the breast from the background. This thresholding step assumes that the mammographic image contains only two classes of pixels, and it calculates the optimal threshold separating the two classes in such a way that their intraclass variance is minimized. This step results in a binary image containing only foreground and background pixels. In the third step, a morphologic opening filter with a circular structuring element is applied to the binary picture to reduce image artifacts and eliminate falsely classified pixels. As a result, the binary image consists of several separated foreground components, including the breast itself and the x-ray film labels. In the last step, the largest connected component is determined, as it is assumed that this segment is the breast region. All of the other components are discarded and erased, resulting in an image containing the breast contour. After visual checking of every delineation (by KH and CRL), manual correction of the breast segmentation had to be carried out in approximately 10% of the images.

### Image analysis

In total, 470 features were calculated to characterize the mammographic images in the present study. The features were selected on the basis of a study that compared various methods of texture analysis and applied them to reference images from publicly available databases, such as the Brodatz, Tilda, and VisTex databases [27,28]. The texture analysis methods chosen for the present investigation correspond to those that had the highest recognition rates in the study; they are described briefly later. They comprise features that are calculated only from the gray-level values (first-order statistics) or from comparison of pixels with defined spatial relations (second-order statistics).

*Statistical features *are calculated from the gray-level values and consist of histograms, gray-level co-occurrence matrices (GLCMs), and sum and difference histograms (SDHs). The full spectrum of all 256 gray levels is divided into 16 categories. The frequencies of the pixels in each category are called histogram features. In addition, frequencies are calculated for the sums and differences of the gray-level values of pixel pairs with defined spatial relations. These texture features are referred to as SDHs [29]. GCLMs are constructed by comparing the gray-level values for two pixels with a defined spatial relation. Combination frequencies of occurrence are calculated for each possible gray-level value. The frequencies in the GCLMs are used to calculate 13 different features [30].

*Moment-based features *are calculated from the pure gray-level values in the ROI and include mean, variance, skewness, and kurtosis, for example. These four features are referred to as central moments (CMs). In addition, the moment-based features are normalized relative to the position of each single pixel within the ROI, resulting in 16 normalized central moments (NCMs). Hu and Zernike [31-33] later proposed transformations of the NCMs to make the results invariant relative to the orientation of the ROI. This resulted in seven invariant moments in the Hu method and 49 moments in the Zernike method.

*Form-based features *are related to the delineated geometric breast area with a closed boundary. They describe only the shape of the ROI, without taking into account the gray-level distribution inside the enclosed area. These features include area, perimeter, compactness, rectangularity, and circularity. Additional features are the normalized radial length and Fourier descriptors to characterize the border shape. Moments based on the binary picture of the breast versus the background are also computed, describing the morphologic appearance of the ROI. Specifically, these features include normalized central moments, Hu moments, and Zernike moments.

*Structural features *are used to obtain information about the structure of the microtexture. Chen *et al. *[34,35] proposed computation of 16 features from the geometric properties of connected regions with similar gray-level values in a set of binary images, known as statistical-geometric features (SGFs). Run-length (RL) features [36] are a similar approach, combining geometric and statistical aspects and describing the microtexture by counting consecutive, collinear pixels ("runs") with the same gray-level values. These features are obtained from a matrix containing the number of runs for each gray level and are computed for four directions.

*Spectral features *characterize textured image regions that show periodic structures, which lead to local maximums at the respective frequencies in the Fourier spectrum. Similarly textured regions thus show similar frequency spectra [37]. We use the wavelet transform [38] to decompose an image iteratively into four components based on frequency content and orientation. For each subcomponent, a feature is computed describing its energy.

For the features based on GLCMs, SDH features, and structural features, additional features were calculated that were based not on single pixels, but on coarser resolutions in the mammogram (0.5 × 0.5 cm and 1 × 1 cm).

### Statistical analysis

The cases and controls were matched 2:1 by age at the time of mammography (within deciles). The study population was randomly divided into a training set (433 cases and 210 controls) and a validation set (431 cases and 208 controls), while retaining the matched nature of the data.

As many of the 470 features turned out to be highly correlated, 128 features with Spearman correlations > 0.98 were excluded from further analyses, leaving 342 features used.

Logistic regression analyses were carried out with these 342 preselected features to identify features that were associated with breast cancer case-control status. Analyses were initially carried out within each group of features (moment-based, form-based, statistical, structural, and spectral). Later, analyses were done across the feature groups.

Five hundred bootstrap samples of the same size as the training set were selected with replacement from the training set. For each bootstrap sample, a stepwise backward logistic model selection procedure, starting with all the features of a specific feature group, was carried out to obtain the best model according to the Akaike information criterion. The features retained from each bootstrap sample were recorded, and a final variable selection was made by applying a procedure proposed by Sauerbrei and Schumacher [39] to this setting. In this procedure, the most frequent features (> 70%) were selected, and due to correlations among some features, the feature with the larger frequency of each highly frequent pair of features (> 90%) was chosen. A multiple logistic regression model using these finally selected features was fitted with the training data.

A score of between 0 and 100 (percent) was assigned to each subject (case or control) in the validation data set. The inverse logit of the linear combination of the subject's measurements in the validation data with the regression coefficients and the intercept coefficient estimated by the previously mentioned multiple logistic regression model was taken as the score value. In other words, multidimensional data points were mapped onto a one-dimensional space by applying the regression coefficients, estimated from the training data set, to the measurements of the corresponding features in the validation data set.

The score was used in a simple logistic regression model for the unadjusted analysis and in multiple logistic regression models for the adjusted analyses. Odds ratios (ORs) and the area under the curve (AUC) of the receiver operating characteristic were calculated to compare the predictive strengths of the feature groups. To study the additional value of the feature groups for predicting case-control status, these *feature group scores *were applied in multiple logistic regression models along with the well-known risk factors of percentage mammographic density (PMD), body mass index (BMI), age at the time of mammography, parity, family history of breast cancer, and age at first term pregnancy as adjusting variables. For purposes of comparison, the adjusting factors were chosen in the same way as in Manduca *et al. *[19] and in the previous case-control study [25].

The same variable selection procedure as described earlier was used to obtain the strongest features across the feature groups, starting with a combination of all of the selected features within the groups. A score was constructed again (called *the final feature score*), and its predictive power was studied by using logistic regression models.

To avoid overfitting, all model selection procedures were carried out with the training data, and the models (particularly the five feature group scores and the final feature score) were validated by using a separate validation data set. Repetitive variable selections were carried out to stabilize the stepwise regression results [40].

All of the tests were two-sided, and a *P*-value of < 0.05 was regarded as statistically significant. Calculations were carried out by using the R system for statistical computing (version 2.11.1; R Development Core Team, Vienna, Austria, 2010).

## Results

The characteristics of the patients included in the study are shown in Table 1. Cases and controls were age matched; the cases had a higher BMI (*P *< 0.00001; t-test), lower parity (*P *< 0.01; Wilcoxon test), and had a family history of breast cancer in a first-degree relative less frequently (*P *= 0.03; χ^2 ^test). In addition, the cases had a higher average age at last menstruation (*P *< 0.01; t-test) and were receiving hormone replacement therapy more often (*P *< 0.00001; χ^2 ^test). No significant differences were found between cases and controls with regard to the other characteristics.

**Table 1:** Characteristics of the study population relative to case and control status

Characteristic	Cases (*n *= 864) Mean (± SD) or count (%)	Controls (*n *= 418) Mean (± SD) or count (%)
Age at mammogram (years)	57.5 (± 10.8)	57.3 (± 10.6)
BMI (kg/m^2^)	26.1 (± 5.0)	24.6 (± 3.8)
Age at last menstruation {years}	48.7 (± 5.5)	47.5 (± 6.6)
Age at first menarche (years)	13.5 (± 1.6)	13.4 (± 1.4)
Age at FTP (years)	25.2 (± 4.6)	25.6 (± 4.4)
Menopausal status		
Premenopausal	221 (30.9%)	105 (26.6%)
Postmenopausal	495 (69.1%)	209 (73.4%)
Parity		
No birth	125 (15.5%)	54 (14.3%)
1 to 2 births	507 (62.7%)	202 (53.4%)
≥ 3 births	177 (21.9%)	122 (32.3%)
Family history of breast cancer		
No	700 (85.6%)	237 (80.3%)
Yes	118 (14.4%)	58 (19.7%)
HRT ever		
No	564 (70.2%)	156 (42.5%)
Yes	239 (29.8%)	211 (57.5%)

For the cases, age is identical with age at diagnosis. BMI, body mass index; FTP, first term pregnancy; HRT, hormone replacement therapy.

Table 2 shows the variable selection process, described in the Methods section, which was used to identify features associated with breast cancer case-control status. From the set of 342 preselected texture features, the selection process within feature groups yielded 99 features (eight moment-based, 16 form-based, 46 statistical, 23 structural, and six spectral), with which five feature group scores were constructed. Building a final model across all feature groups starting with these 99 features resulted in the inclusion of 46 features in the final feature score (one moment-based, four form-based, 29 statistical, 10 structural, and two spectral).

**Table 2:** The process of variable selection

Feature group	Total features	**Preselected features**^**a**^	Selected features **within feature group**^**b**^	Features **finally selected**^**c**^
Moment-based features	76	71	8	1
Form-based features	86	74	16	4
Statistical features	130	86	46	29
Structural features	108	90	23	10
Spectral features	70	21	6	2
Total	470	342	99	46

Numbers of features are shown. ^a^Preselection due to high correlations between features. ^b^Feature group scores were constructed with these features. ^c^The final score was constructed with these features.

Table 3 shows the main results for PMD and the selected sets of the texture features. The selected features within each group were more predictive than PMD in both the unadjusted analysis and the adjusted analyses. In the validation data set, the AUCs of the simple regression models with the feature group scores as the only independent variable ranged from 0.58 (moment-based features) to 0.72 (statistical features), whereas the AUC was 0.51 for the PMD model. Consequently, the odds ratio per standard deviation (SD) change (that is, per interval of length SD) was larger for the feature group scores (between 1.46 and 2.40) than that of the PMD model (1.05). Including epidemiologic risk factors such as BMI, parity, family history, and age at first term pregnancy in the models did not change or even strengthened the AUCs, and all of the ORs for the feature group scores remained significant. The AUC of the fully adjusted multiple models within the feature groups ranged from 0.67 to 0.74 (again, moment-based and statistical features, respectively). In this setting, the AUC for the PMD model (0.66) was again lower than the AUCs for the feature groups, and was slightly higher than the AUC for the regression model with risk factors alone (0.65).

**Table 3:** Simple and multiple logistic regression models to measure the predictive power of percentage mammographic density (PMD) and selected features within and across the five texture feature groups, via feature group scores and the final feature score, respectively^a^

	Training data set		Validation data set				
	
		Unadjusted		Adjusted for age and BMI		Adjusted for age, BMI, parity, family history, and age at FTP	
		
Texture features included	AUC	AUC	OR (95% CI)	AUC	OR (95% CI)	AUC	OR (95% CI)
None^b^	-	-	-	0.60	-	0.65	-
PMD	0.53	0.51	1.05 (0.89-1.23)	0.61	1.24 (1.00-1.55)	0.66	1.19 (0.93-1.53)
Moment-based features (*n *= 8, group 1)	0.66	0.58	1.46 (1.22-1.73)	0.62	1.43 (1.19-1.72)	0.67	1.41 (1.14-1.75)
Form-based features (*n *= 16)	0.67	0.59	1.47 (1.23-1.74)	0.64	1.44 (1.20-1.74)	0.67	1.49 (1.21-1.84)
Statistical features (*n *= 46)	0.82	0.72	2.40 (1.98-2.90)	0.73	2.28 (1.87-2.78)	0.74	2.36 (1.88-2.96)
Structural features (*n *= 23)	0.77	0.65	1.64 (1.38-1.95)	0.68	1.60 (1.34-1.92)	0.71	1.70 (1.39-2.08)
Spectral features (*n *= 6)	0.71	0.65	1.67 (1.40-1.99)	0.67	1.57 (1.30-1.90)	0.68	1.60 (1.29-1.98)
Selected features across all feature groups (final model; *n *= 46)	0.85	0.75	2.65 (2.18-3.21)	0.75	2.55 (2.08-3.11)	0.79	2.88 (2.28-3.65)
Selected features across all feature groups + PMD	0.85	0.75	2.63 (2.17-3.18)	0.75	2.52 (2.06-3.08)	0.79	2.86 (2.26-3.62)

The area under the curve (AUC) of the regression models and the odds ratio (OR) per standard-deviation (SD) change for the feature scores with 95% confidence intervals are shown. Features were selected as described in the Patients and Methods sections.

AUC, area under the curve; BMI, body mass index; CI, confidence interval; FFTP, first term pregnancy; OR, odds ratio; PMD, percentage mammographic density; SD, standard deviation. ^a ^In the training data, each logistic regression model used selected features as independent variables; in validation data, the logistic regression models used the feature group scores and the final feature score, respectively, as independent variable. Adjusted analyses with regular risk factors as additional independent variables. ^b^Prediction only with regular risk factors.

The texture features finally selected across all feature groups improved the predictive power in all of the analyses. An AUC of 0.79 with an OR per SD change of 2.88 (95% CI, 2.28 to 3.65) was reached with the final score on the validation data. Only small differences were noted between the unadjusted and adjusted analyses. Additional inclusion of the percentage density did not lead to any improvement in the model (AUC, 0.79; OR, 2.86; 95% CI, 2.26 to 3.62).

The final score was tested separately in analogue mammograms and digital mammograms. The AUC for the analogue images was larger, at 0.84 (fully adjusted model) than the AUC for the entire data set, whereas the AUC of 0.76 (fully adjusted model) for the digital mammograms was smaller than the AUC for the entire data set.

**Figure 1:**
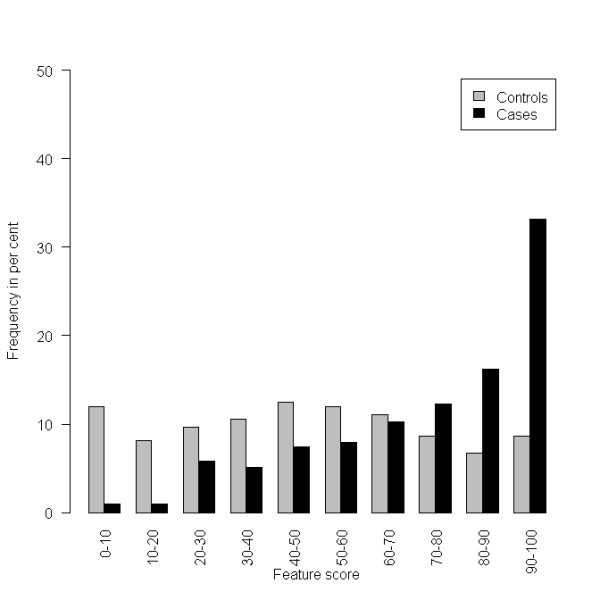
**Histogram of the final feature score, based on the 46 finally selected features applied on the validation data set**.

**Figure 2:**
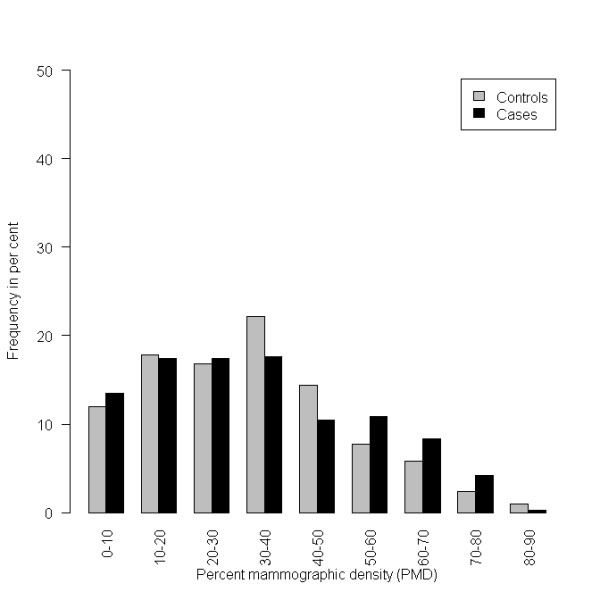
**Histogram of the percentage mammographic density (PMD) on the validation data set**.

The distribution of the score in the final model built from the texture features is shown in Figure 1, and the distribution of the PMD is shown in Figure 2. The mammographic density shows an expected distribution, many score values of breast cancer patients are at the higher end of the scale.

**Figure 3:**
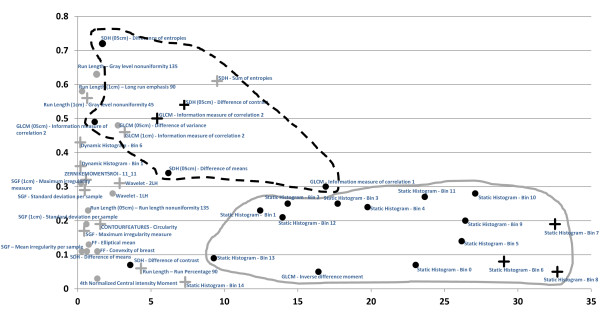
**Finally selected features (*n *= 46)**. Strength of risk prediction within the final logistic regression model on x-axis (absolute value of log odds ratio per standard deviation) and the feature's Spearman correlation with percentage mammographic density (PMD) on the y-axis. ^+^The texture feature and PMD have the same direction with regard to their association with risk (that is, either positive log OR and positive correlation with PMD or negative log OR and negative correlation with PMD). ^•^The texture feature and PMD have the opposite direction with regard to their association with risk (that is, either positive log OR and negative correlation or negative log OR and positive correlation with PMD). Gray symbols, Feature is selected in fewer than 90% of the bootstrap samples. Black symbols, It is selected in more than 90% of the bootstrap samples. The dashed line circumscribes a cluster of second-order statistical features, and the continuous gray line circumscribes a cluster of first-order statistical features. "Static histogram" refers to features describing the relative frequency of gray-level values according to a given interval (bin). These features are thus first-order statistics describing the gray-level distribution. SDH refers to features calculated from sum and difference histograms, and GLCM refers to features calculated from a gray level co-occurrence matrix. Both of these are second-order statistics, describing the gray-level distribution relative to spatial relations between adjacent pixels. SGF refers to the statistical geometric features, describing the structure of the microtexture. A more-detailed description of all of the features is given in the Methods section.

The features finally selected are shown in Figure 3. The absolute value of each feature's regression coefficient in the final logistic regression model was plotted against the absolute value of the feature's correlation with PMD. It was noted whether these features were selected in more than 90% of the bootstrap repetitions and whether the direction of the risk association was the same as in the mammographic density.

**Figure 4:**
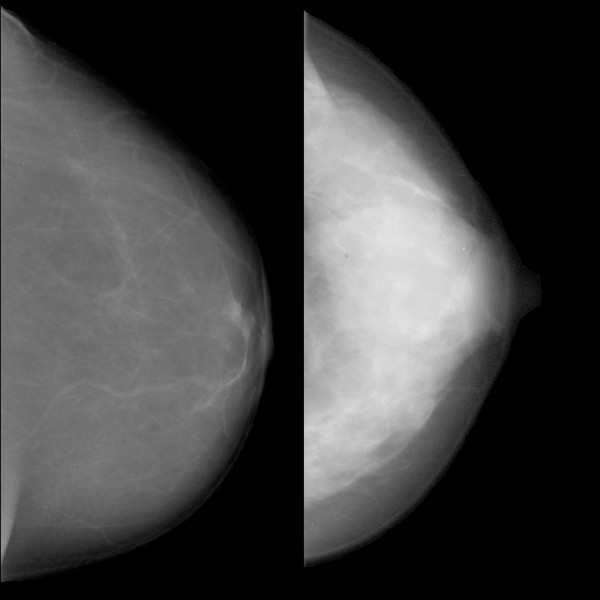
**Example of a feature with the same direction for the correlation of the feature with breast cancer risk and percentage mammographic density (PMD)**. Patients with mammograms like that on the left had low values for the feature "SDH (0.5 cm) difference of contrast" and had a low predicted risk of breast cancer. Patients with mammograms like that on the right had high feature values, a high risk of breast cancer, and a high mammographic density. The Spearman correlation with PMD for this feature was +0.54.

**Figure 5:**
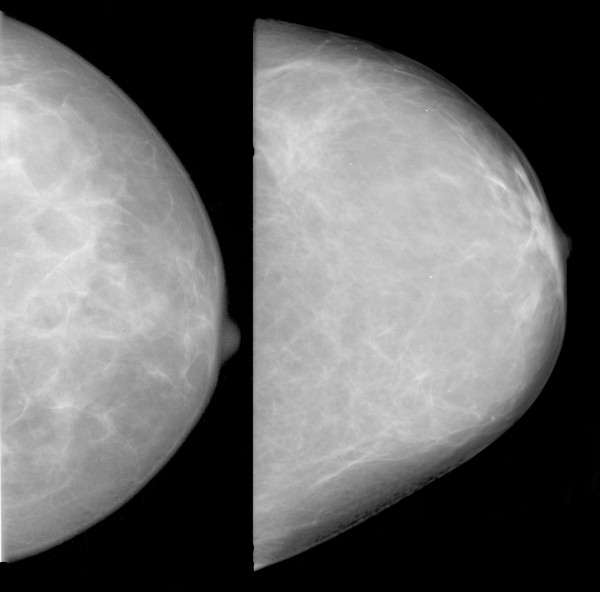
**Example of a feature with no correlation with percentage mammographic density (PMD)**. Patients with mammograms like that on the left had low values for the feature "GLCM inverse difference moment" and had a low predicted risk of breast cancer. Patients with mammograms like that on the right had high feature values and a high risk of breast cancer. The Spearman correlation with PMD for this feature was -0.05.

**Figure 6:**
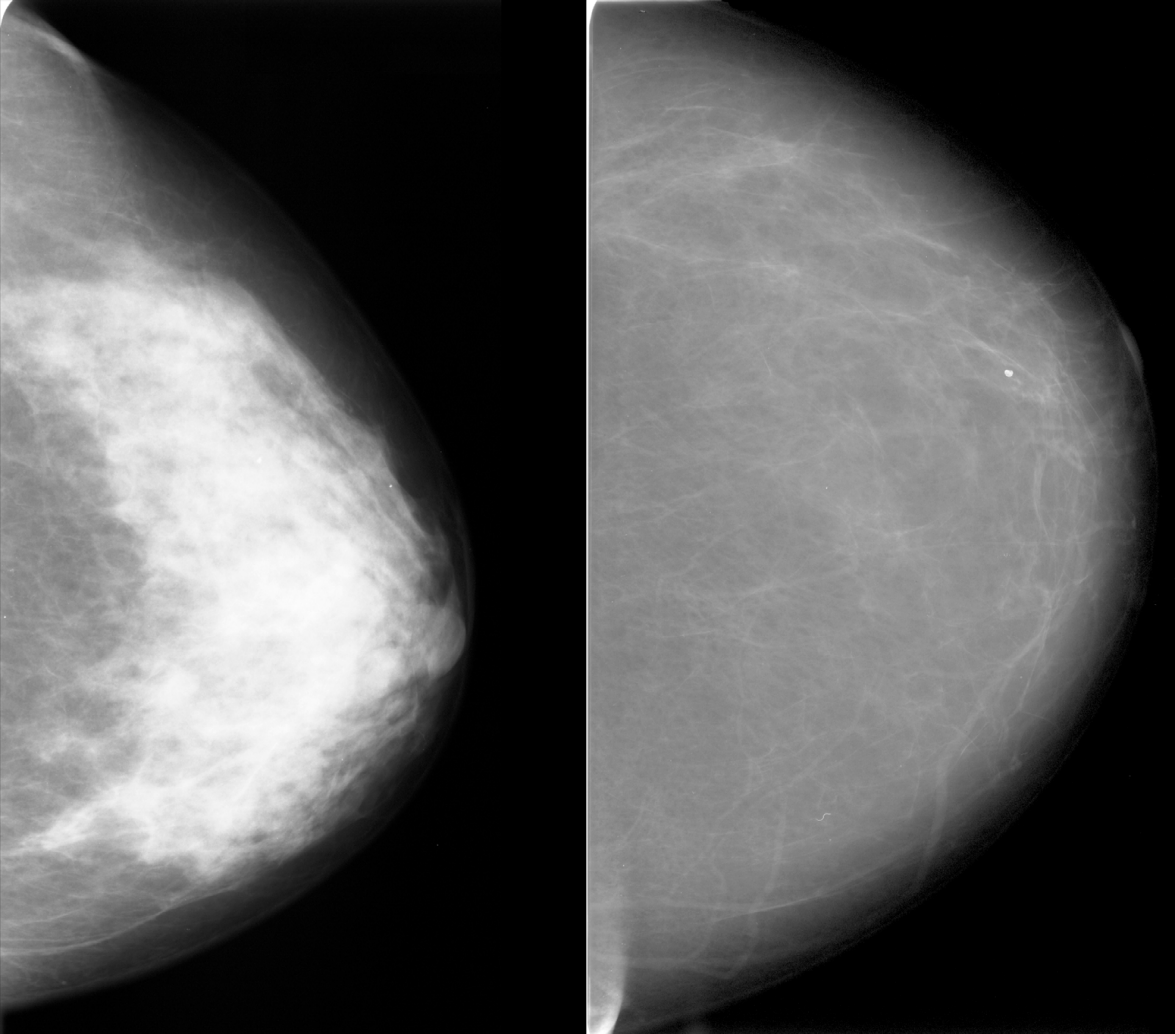
**Example of a feature with different directions for the correlation with breast cancer risk and PMD**. Patients with mammograms like that on the left had low values for the feature "SDH (0.5 cm) difference of entropies" and had a low predicted risk of breast cancer and a high mammographic density. Patients with mammograms like that on the right had high feature values, a high risk of breast cancer, and a low mammographic density. The Spearman correlation with PMD for this feature was -0.72.

**Figure 7:**
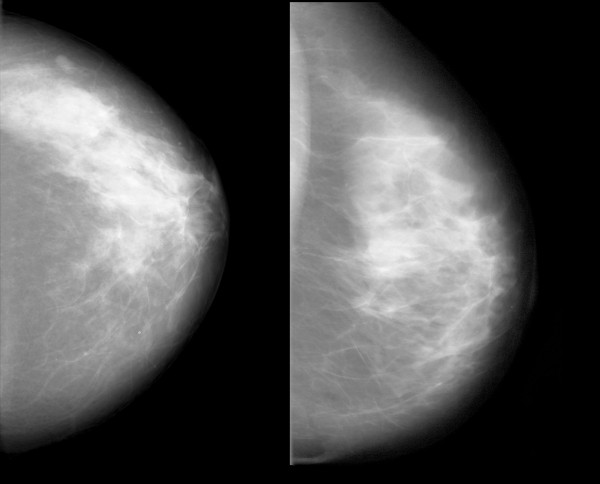
**Examples of images with low score values calculated with the final prediction model and a low risk of breast cancer (left), and images with high score values and a high risk of breast cancer (right)**. Spearman's rho for the correlation between the final score and percentage mammographic density (PMD) was 0.02.

Some features correlated with risk in the same direction that they correlated with mammographic density; an example is shown in Figure 4. Some features did not correlate with PMD, but had high regression coefficients. Visual inspection did not reveal any known anatomic characteristics that correlated with the value of the feature. An example of this type of feature is given in Figure 5. For some features, the associations with PMD and breast cancer risk were in inverse directions (Figure 6). Examples of mammograms with high and low score values are presented in Figure 7. No correlation was found between the score values and PMD.

Statistical features show above-average representation in the final model. They make up 29 of the 46 final features and provide all of the features that are selected in more than 90% of variable selection repetitions (black symbols in Figure 3). Statistical features clustered into two different groups: one with a strong association with PMD and lower coefficient values in the final score model (GLCM and SDH), and the other with a weaker association with PMD and high coefficient values in the final score model.

Ten of the 12 features in the first group show a strong correlation with PMD (Spearman's ρ, 0.30 to 0.72). The one with the highest correlation with PMD, "SDH (0.5 cm) difference of entropies", played a minor role in the final score model because of its low regression coefficient. This feature describes the entropy (a measure of information) of the difference histogram on a coarse version of the image. High values indicate an ROI containing a variety of inhomogeneous patterns, whereas low values correspond to a uniform ROI. Two other features from this group, "GLCM, correlation measure type 1", which is a weighted version of the entropy measure, and "GLCM, inverse difference moment", which describes the distribution of areas with high local contrast between fatty and dense tissue, played a major role in the prediction model. In the latter feature, low values indicate increased local contrast, corresponding to a lower risk, whereas high feature values indicate the opposite.

The latter group consists mainly of 15 histogram features that together represent the whole spectrum of all gray scales in the mammogram. Those that refer to gray levels in the middle range (bin 6 to 8) have the highest coefficients, are positively associated with PMD, and have the same direction of association with risk as PMD. However, the correlation with PMD is rather weak (Spearman's ρ, 0.05 to 0.19).

Features from other groups, such as structural features or form-based or spectral features, were less likely to be selected for the final prediction model. They range much closer to the y-axis in Figure 3, and only some of them appear to correlate with PMD.

## Discussion

In this breast cancer case-control study, a statistical model was constructed that is able to predict case-control status by using image-texture analysis features that were calculated automatically from areas of breast in digitized mammograms. Adding the percentage mammographic density to a risk model using the automated texture features did not improve risk prediction in this study.

As in other studies of automated image-texture analysis [41-45], the texture features examined consisted of first-order features such as gray-level distributions, the computation and distribution of the spatial relations of gray-level values from second-order statistics, one run-length measure, and spectral frequency measures obtained from the wavelet transform. In contrast to other studies, the statistical evaluation used in the present investigation also selected additional features describing the contour and form of the marked breast area, for example, as well as structural measures from the statistical-geometrical features (SGFs) suggested by Chen *et al. *[34,35]. Specifically, SGFs describe the microstructure of the breast tissue, providing high contrast between the breast and the involuted surrounding tissue. Whereas Manduca *et al. *[19] specifically computed the textural measures they used within a constant-thickness region (CTR), which they defined as an area approximately 160 pixels inside the perimeter of the breast region, the present study used the complete breast tissue delineated to calculate the various texture features. It is not possible on the basis of the present study to determine whether one of these approaches is better than the other.

The model-building methods (that is, separate training and validation data sets and bootstrap resampling procedures, along with stepwise model selection) are comparable with those used in an earlier study [19]. Contrary to that study, multifactorial models were finally used to predict the case-control status.

To investigate the visual meaning and biologic nature of the 46 features finally selected, the feature values were compared with the assessed PMD, and the corresponding mammograms were inspected visually. Features that were expected to show similar texture characteristics, such as gray-level frequencies or GLCM and SDH, are clustered together in Figure 3. Some other features are clustered close to the y-axis (corresponding to a lower predictive value in the final model) and belong to specific feature groups, such as spectral or form-based features.

Most of the features finally selected were statistical features. Some of these (GLCM and SDH) are associated with PMD. The nature of GLCMs makes it clear why most of the features obtained may represent the PMD: variations and differences in intensities and texture in the image being examined are directly reflected in the GLCM [29]. The fact that most of the GLCM and SDH features are clustered together in Figure 3 confirms what was predicted hypothetically [29].

The other statistical features are gray-level value intervals, representing features that all have a poor association with PMD. However, it might be hypothesized that these features together describe dense and nondense areas in the mammogram and that they might jointly provide information that would be similar to mammographic density.

To assess textural structures at various levels, the statistical features were computed on three different scales of the image: the original mammograms and two reduced versions of the mammograms downscaled to pixel sizes of 0.5 cm and 1.0 cm per pixel. Interestingly, the 12 second-order statistical features were selected from all three image scales. Specifically, six of the 12 features were computed on the full-resolution image, whereas the other six features were computed on either of the two downscaled versions. This effect shows that visual information is assessed based on fine as well as coarse structures in the breast tissue. The change in the coarseness can sometimes result in impressive changes in the association with PMD, such as "SDH - difference of contrast" (Spearman's ρ = 0.07) and "SDH (0.5 cm) difference of contrast" (Spearman's ρ = 0.54). This might suggest that when one is looking for features that explain mammographic density, one level of coarseness may be best related to mammographic density. Similar observations were made by Manduca *et al. *[19] by using wavelet features; the authors showed that feature assessment resulted in higher AUCs when the texture features were computed on a coarser scale.

As form-based features describe the convexity of the breast (and hence implicitly the stiffness of the tissue during compression in the image-acquisition process), it seems that stiffness does play a role in PMD, but only a minor one. Some structural features were selected that describe small and large connected areas of breast tissue and fat, but the quantity, form, and size of such connected regions appear to be less important. Finally, only two spectral features were included during the selection process, suggesting that periodic structures appear to be present in the ROI, but that they play a minor role in PMD and have almost no effect on the risk score.

### Strengths and weaknesses

In addition to its strengths, a large sample size, the inclusion of a comprehensive set of automatically computed textural features, and robust statistical methods with strict separation of training and validation data, the present study also has some weaknesses. The original case-control study was designed to detect genetic susceptibility factors for breast cancer, and the mammogram study has a recall bias in the control group. Only half of the women in the control group had mammograms. This may have been why the detectable effect in the present study was rather low, with an OR of 2.3 (95% CI, 1.5 to 3.6), in comparison with other published studies. Moreover, some unexpected distributions of risk factors appeared in the study, such as the higher frequency of a family history of breast cancer in the control group. This effect might be explained by volunteer bias, leading to an accumulation of risk factors in the group of volunteer controls. Earlier studies by our group have shown that awareness of the risk of breast cancer leads to greater willingness among women to address their own risk of breast cancer, either by obtaining information about the risk or by taking part in chemoprevention studies [46,47]. However, all of these imbalances in the frequency of risk factors were adjusted for.

Trying to translate the use of texture features into risk assessment for the patients, it is not clear how helpful this approach will be to correlate this risk assessment with patient or tumor biology. When mammographic density is compared with texture features, it appears to be clear that in the context of risk prediction, mammographic density is closely associated with a biologic correlate. Although the precise composition of tissue that is responsible for mammographic density has not yet been fully understood, several biologic effects can be regarded as logical. Hormone exposure, for example, increases breast density and also the risk of breast cancer. Mammographic density changes throughout life and reacts to hormone exposure. By contrast, the texture features in the analysis presented here were selected on the basis of their ability to differentiate between the mammograms of breast cancer patients and healthy controls, resulting in a mathematical model that may not be easily anticipated by the human brain or its visual functions. The interaction between image features that results in the differentiation could be a complex one and definitely needs further exploration.

Another concern in the present study might be that digital mammograms were handled in the same way as analogue ones, as standards for assessing digital mammograms are still pending. However, a recent study found a high degree of correspondence between textural features in digital and analogue mammograms [48]. In the present study, it was found that the final score is useful in digital mammograms, although their predictive value is lower than that in analogue images.

## Conclusions

The present study has shown that texture-analysis features may be helpful in predicting the risk of breast cancer. It is too early for conclusions to be drawn from these findings regarding the feasibility of the method used here for other study groups. Differences in mammographic imaging methods and in standardizing the production and processing of images may have led to results that are highly specific to the present study. A standardized stock of texture-analysis features that could be applied independently in other studies is not yet available.

However, because adding percentage mammographic density to the final score model did not improve the model's predictive power, and as some features appear to represent mammographic density and others appear to be independent of it, further research is warranted to investigate the additional predictive value of these analysis tools.

## Abbreviations

AUC: area under the curve; BMI: body mass index; CI: confidence interval; CM: central moment; CTR: constant-thickness region; FTP: first term pregnancy; GLCM: gray-level co-occurrence matrix; HRT: hormone replacement therapy; MD: mammographic density; NCM: normalized central moment; OR: odds ratio; PMD: percentage mammographic density; RL: run-length; ROI: region of interest; SD: standard deviation; SDH: sum and difference histogram; SGF: statistical-geometric feature.

## Competing interests

The authors declare that they have no competing interests.

## Authors' contributions

LH carried out the statistical analysis, wrote parts of the article, and revised it. FW carried out computer analysis of the images and wrote parts of the article. PAF medically interpreted the results, wrote parts of the article, and revised it. SMJ provided the mammographic density measurements and coordinated the acquisition of the mammograms. KH and CRL carried out mammographic density measurements. AH and CD contributed clinical information. CH carried out mammographic density measurements. MPL contributed clinical information. KB carried out parts of the mammogram acquisition. ME and CM designed the computer analysis. RS, MMM, and BRA carried out clinical assessment of the mammograms. MU provided the infrastructure for mammogram acquisition. MWB revised the article. TW wrote parts of the article and carried out computer analysis of the images. All of the authors read and approved the final manuscript.
